# Family and Neighbourhood Socioeconomic Inequalities in Childhood Trajectories of BMI and Overweight: Longitudinal Study of Australian Children

**DOI:** 10.1371/journal.pone.0069676

**Published:** 2013-07-23

**Authors:** Pauline W. Jansen, Fiona K. Mensah, Jan M. Nicholson, Melissa Wake

**Affiliations:** 1 Murdoch Children’s Research Institute, Erasmus MC-University Medical Center Rotterdam, Rotterdam, The Netherlands; 2 Department of Child and Adolescent Psychiatry/Psychology, Erasmus MC-University Medical Center Rotterdam, Rotterdam, The Netherlands; 3 Clinical Epidemiology and Biostatistics Unit, Royal Children’s Hospital, Melbourne, Australia; 4 Department of Pediatrics, University of Melbourne, Melbourne, Australia; 5 Parenting Research Centre, Royal Children’s Hospital, Melbourne, Australia; 6 Centre for Community Child Health, Royal Children’s Hospital, Melbourne, Australia; University of Swansea, United Kingdom

## Abstract

**Background:**

Socioeconomic inequalities in longitudinal patterning of childhood overweight could cause marked differentials in total burden by adulthood. This study aims to determine timing and strength of the association between socioeconomic status (SES) and children’s body mass index (BMI) in the pre- and primary school years, and to examine socioeconomic differences in overweight trajectories across childhood.

**Methods:**

Participants were 4949 children from the Longitudinal Study of Australian Children. BMI was measured at four biennial waves starting at age 4–5 years in 2004. Developmental trajectories of childhood overweight were identified with latent class analyses. Composite variables of family and neighbourhood SES were used.

**Results:**

Socioeconomic differences in mean BMI z-scores already present at age 4–5 more than doubled by age 10–11 years, reflecting decreasing mean BMI among advantaged rather than increasing means among disadvantaged children. Latent class analysis identified children with ‘stable normal weight’ (68%), and with ‘persistent’ (15%), ‘late-onset’ (14%), and ‘resolving’ overweight (3%). Risks of persistent and late-onset childhood overweight were highest among low SES families (e.g. most disadvantaged quintile: OR_persistent_ = 2.51, 95%CI: 1.83–3.43), and only partly explained by birth weight and parental overweight. Relationships with neighbourhood SES were weaker and attenuated fully on adjustment for family SES. No socioeconomic gradient was observed for resolving overweight.

**Conclusions:**

Childhood has become the critical period when socioeconomic inequalities in overweight emerge and strengthen. Although targeting disadvantaged children with early overweight must be a top priority, the presence of childhood overweight even among less-disadvantaged families suggests only whole-society approaches will eliminate overweight-associated morbidity.

## Introduction

Overweight is a worldwide public health concern [1] given its adverse immediate and long-term consequences [2], [3]. Overweight is more prevalent among adults from socially disadvantaged backgrounds and neighbourhoods [4], [5]. Large socioeconomic inequalities are also observed in prevalence of the morbidities associated with overweight and obesity, such as cardiovascular disease and diabetes [6], [7].

Social gradients in prevalence of childhood and adolescent obesity were not apparent before the mid-1990s [8], [9] but, as the obesity epidemic evolved, inequalities rapidly developed [10] and widened, even while the overall prevalence of overweight/obesity has begun to stabilize [11], [12]. Thus, current generations of children from low socioeconomic backgrounds may well demonstrate even wider socioeconomic inequalities in adult obesity and associated morbidities than do adults today [6], [7]. Nonetheless, burden will continue to be experienced – even if unequally – across all social classes because even the most advantaged now suffer obesity levels that were unprecedented 30 years ago. Tackling overweight effectively is likely to require a ‘proportionate universalism’ approach whereby interventions are implemented at a level according to their need across the social gradient [13]. To do this requires a much clearer understanding of patterns of disproportionate need across the SES gradient, showing not only when in life socioeconomic inequalities in overweight emerge, but also how and why they persist during childhood.

Large British and Canadian studies have recently observed socioeconomic differentials in overweight beginning to emerge early in childhood, widening until late childhood [14], [15], then remaining stable over the adolescent years [16]. Four American [17]–[19] and Canadian [20] studies, focusing on *heterogeneity* as opposed to *timing* of children’s growth patterns, have identified distinct weight trajectories across childhood, either based on continuous BMI or overweight status. Common to all four studies was a ‘late-onset overweight’ trajectory, characterized by children gradually becoming overweight during childhood [17]–[20], while two studies [17], [19] also identified an ‘early onset of persistent overweight’ trajectory. Various indicators of low family SES, e.g. low parental education and insufficient income, were identified as risk factors for both trajectories, particularly late-onset overweight [17]–[20].

However, there remain a number of important unknowns. These studies examined family but not neighbourhood disadvantage [17]–[20], while investigation of both might clarify the nature of disadvantage that impacts most on unhealthy weight trajectories. Family SES influences children’s direct eating environment, life-styles and daily routines via intergenerational transmission of health behaviours and eating habits [21], [22]. Arguably, disadvantaged neighbourhoods influence weight in different ways, for instance by availability and accessibility of services and facilities [23]. Furthermore, inequalities may continue to evolve rapidly as more advantaged individuals and communities adapt to limit the ‘obesogenicity’ of their own environments, thereby increasing and entrenching gradients adversely affecting the disadvantaged. If this is the case, we should begin to see ‘resolving early overweight’ trajectories that cluster within the most advantaged. Finally, it remains unclear whether observed gradients persist over and above contributions of key known intermediaries – like parental anthropometry [4], [5], [24] and children’s birth weight [25], [26] – that themselves show strong social patterning and associations with overweight.

The nationally-representative Longitudinal Study of Australian Children [27] offers an ideal opportunity to address these important evidence gaps for a contemporary cohort. Four biennial assessments of measured BMI are available for children born in 1999–2000, well after the obesity epidemic was established and socioeconomic gradients started to become apparent. Children were aged 4–5 years at the start of the study and 10–11 years at follow-up. This is an important period from both a clinical and public health perspective. At the individual level, prediction of adult BMI from BMI in childhood is weak for toddler and early childhood BMI, but becomes much stronger with increasing age [28], [29]. At the population level, the first point of systematic BMI screening/surveillance in many countries is close to the transition to primary school at around age 5 years [30], [31]. Thus, understanding how socioeconomic status affects typical BMI trajectories children follow from that age could influence public health responses to these population surveillance data.

In this paper, we first investigate how the cross-sectional SES-BMI association develops during the pre- and primary-school period. In line with the British and Canadian findings [14], [15]. we hypothesized that the cross-sectional association between family and neighbourhood SES and BMI already reported to emerge by age 4–5 years in these Australian children [32] would persist and strengthen by age 10–11 years. Our second aim is to examine whether family and neighbourhood SES predict weight trajectories across childhood, as identified with latent class analysis. We present findings for trajectories based on a variety of BMI classifications (overweight, obesity, raw BMI, and BMI z-scores), to explore the robustness of any BMI-SES associations and to facilitate cross-cohort comparisons (often precluded between earlier papers by different choices of BMI classification). Moreover, as latent class analysis groups children on the basis of statistical similarity and thus can be influenced by the format of provided variables, classifying children based on different BMI measures has the potential to produce different classifications with different clinical and public health meaning. We expected that family and neighbourhood SES are independently associated with unhealthy weight trajectories throughout childhood, and that part of this relationship is explained by parental anthropometry and the child’s birth weight.

## Methods

### Study Population

This study was conducted using data from Waves 1 to 4 of the Longitudinal Study of Australian Children (LSAC) [27]. Participants (n = 4983) were aged 4–5 years when recruited in 2004, and 10–11 years (n = 4169, 84% retention) at Wave 4 of data collection in 2010. Data were collected every two years with direct anthropometric measurements and parental questionnaires.

Cross-sectional analyses were conducted in children with complete data on both BMI and SES within a wave. Longitudinal analyses were conducted in 4949 children, having excluded participants without BMI data in any of the four waves (n = 16) and those without information on SES in Wave 1 (n = 18).

### Ethics Statement

This study was conducted in accordance with the principles expressed in the World Medical Association Declaration of Helsinki and has been approved by the Australian Institute of Family Studies Ethics Committee. Parents or legal guardians provided written informed consent for their participating child at the first wave of data collection.

### Measures

#### Body mass index

During home visits at each wave, interviewers measured children’s weight and height using standardized equipment and procedures. After converting BMI (kg/m^2^) into age- and gender-specific z-scores (BMIz) [33], children were classified as ‘normal weight’ (including underweight) or ‘overweight’ (including obesity) by International Obesity Task Force criteria [34].

#### Socioeconomic status

At each wave, LSAC releases composite indicators of family and neighbourhood SES, both of which were categorized into internal quintiles ranging from 20% most disadvantaged to 20% most advantaged families. These composite variables have been validated against other proxies of socioeconomic disadvantage and were associated with adverse outcomes that are often highly socially patterned [35]–[38]. The family SES variable is a standardized summary measure (mean = 0, SD = 1) of parent reports of equivalised annual family income, years of education, and current or most recent occupational status [35]. Neighbourhood SES is determined by linking the Socioeconomic Indexes for Areas disadvantage index with families’ most recent postcode of residence [36]. This census-based index (national mean = 1000, SD = 100, higher values represent greater advantage) is derived from a variety of social and economic characteristics of individuals within defined geographical areas (about 250 urban dwellings) including country of birth, educational level, employment status, and car and home ownership. It thus summarises the SES of every individual in a neighbourhood, though does not tap into more ‘global’ neighbourhood characteristics such as convenience stores, parks and safety.

#### Covariates

Children’s Indigenous status and whether any language other than English is spoken at home were considered as possible confounders, given their links with children’s weight status [32]. To determine whether any observed gradients were robust to known mediators of childhood overweight, children’s birth weight and self-reported parental BMI (non-overweight, overweight, obese) were included in separate regression models. Information on covariates was obtained by parental questionnaires.

### Statistical Analysis

Survey weights taking account of differential non-response (cross-sectional and longitudinal analyses) and sample attrition (longitudinal analyses) were applied in all analyses [39]. Cross-sectional associations between family or neighbourhood SES quintiles and children’s BMIz at each wave were assessed with linear regression analyses (aim 1). Tests of interaction were conducted to assess whether trends in BMI by age differed between SES quintiles. All analyses were adjusted for confounders.

Next, trajectories of childhood overweight from Wave 1 to 4 were identified by latent class analysis using Mplus version 5 [40]. With this technique children were grouped based on repeated measures of BMI dichotomized into normal weight and overweight, our primary BMI classification for this paper. Estimation was by maximum likelihood with robust standard errors, taking account of missing data by inferring on the basis of available measures. Only children with missing BMI data in all waves were excluded. The number of ‘latent classes’ was determined on the basis of the minimum Bayesian Information Criterion and significant Lo-Mendell-Rubin Likelihood Ratio Test. To account for uncertainty in latent classes, conditional probabilities of group membership provided by Mplus were used to create 50 imputed datasets of group membership [41] which were analyzed with the multiple imputation and survey design facilities of STATA 11.0 (Stata Corporation, College Station, Texas).

To examine socioeconomic differences in the four identified trajectories of childhood weight status (aim 2), we conducted multinomial logistic regression analyses to calculate odds ratios (ORs) for overweight trajectory membership (reference: stable normal weight) per family or neighbourhood SES quintile as measured in Wave 1. Three different models are presented: (1) adjusted for confounding variables; (2) model 1 further adjusted for birth weight and parental BMI as potential mediators; and (3) model 2 including family and neighbourhood SES simultaneously, to obtain SES effect estimates independent of each other. For missing values in maternal (n = 376) and paternal BMI (n = 1182), a missing category was added. Missing values in birth weight (n = 81) were replaced by the sample mean value.

Finally, we re-ran the latent class analyses for (b) trichotomized BMI, (c) continuous BMI raw scores, and (d) continuous BMIz, then repeated the multinomial logistic regression analyses for each of these categorizations to examine socioeconomic differences in trajectories of obesity, raw BMI and BMIz respectively.

## Results

BMI data were available for 4934 children in Wave 1, 4423 children in Wave 2, 4289 children in Wave 3 and 4018 children in Wave 4. In nearly all waves, data on BMI were more often missing among children of families or neighbourhoods with a low socioeconomic status (*P*-values in all waves <0.05, except in Wave 1 *P*-value = 0.14). In total, 3737 children had BMI data at all four waves, 582 at three waves, 295 at two waves, and 335 at one wave.

Of all children included in the analyses, 51% were boys, and 4% and 11% had Indigenous and non-English speaking backgrounds respectively. At Wave 1 when children were 4–5 years old, the majority (80%) were normal weight, 15% overweight, and 5% obese. By age 10–11 years, percentages of children with overweight or obesity increased to 20% and 6%, respectively. Mean neighbourhood SES was 1011 (SD = 59, range: 660–1160), slightly higher than the national population mean of 1000 [42] (t-test = −7.7, *P*<0.001). Children of the most disadvantaged as compared to the most advantaged socioeconomic backgrounds weighed on average 81 grams less at birth (95%CI: −136, −27) and their parents were more likely to be obese (OR_mothers_ = 4.18, 95%CI: 3.10, 5.70; OR_fathers_ = 2.03, 95%CI: 1.49, 2.77).

Cross-sectional analyses showed that mean BMIz was markedly higher for children of low socioeconomic families (Table 1; *P* for SES trend <0.001 at every age). Strikingly, in the most advantaged quintile, mean BMIz fell sharply with age from 0.47 at age 4–5 years to 0.19 by age 10–11 years. In the most disadvantaged quintile, mean BMIz started high (0.65 at age 4–5), decreased only little thereafter, and rose again between ages 8–9 and 10–11 years. Therefore, strong cross-sectional gradients by disadvantage quintiles already present at age 4–5 years strengthened with time. Cross-sectional findings for neighbourhood disadvantage, although less striking, were also statistically strong from age 6–7 years in the 4^th^ and 5^th^ quintiles.

**Table 1:** Cross-sectional associations between socioeconomic status and BMI z-score across childhood.

		Mean BMIz per quintile (SE)		Mean difference in BMIz compared to most advantaged SES quintile^a^				
Age (years)	n	Most advantaged	Most disad-vantaged	2^nd^ quintile	3^rd^ quintile	4^th^ quintile	Most disadvantaged	*P* for SES trend
				β (95% CI)	β (95% CI)	β (95% CI)	β (95% CI)	
**Family SES**								
4–5	4917	0.47 (0.03)	0.65 (0.04)	0.00 (−0.09, 0.08)	0.08 (−0.01, 0.17)	**0.12** (0.03, 0.21)	**0.17** (0.07, 0.27)	<0.001
6–7	4417	0.26 (0.03)	0.49 (0.04)	0.05 (−0.04, 0.14)	**0.18** (0.08, 0.28)	**0.17** (0.07, 0.27)	**0.23** (0.13, 0.33)	<0.001
8–9	4285	0.23 (0.03)	0.57 (0.05)	0.07 (−0.02, 0.16)	**0.18** (0.08, 0.27)	**0.26** (0.16, 0.36)	**0.33** (0.22, 0.44)	<0.001
10–11	3838	0.19 (0.04)	0.63 (0.05)	**0.10** (0.00, 0.20)	**0.24** (0.14, 0.33)	**0.24** (0.14, 0.35)	**0.44** (0.32, 0.56)	<0.001
*P* for interaction (Age*SES)^b^				0.08	0.01	0.01	<0.001	
**Neighbourhood SES**								
4–5	4934	0.48 (0.04)	0.59 (0.04)	0.04 (−0.07, 0.16)	0.08 (−0.02, 0.19)	0.07 (−0.04, 0.18)	0.10 (−0.01, 0.22)	0.08
6–7	4423	0.26 (0.03)	0.49 (0.04)	0.08 (−0.02, 0.19)	0.07 (−0.03, 0.17)	**0.20** (0.11, 0.29)	**0.22** (0.12, 0.33)	<0.001
8–9	4289	0.29 (0.04)	0.52 (0.04)	0.06 (−0.04, 0.16)	0.03 (−0.07, 0.13)	**0.21** (0.11, 0.31)	**0.23** (0.12, 0.33)	<0.001
10–11	4017	0.26 (0.04)	0.51 (0.04)	0.07 (−0.04, 0.18)	0.09 (−0.02, 0.20)	**0.24** (0.13, 0.35)	**0.25** (0.14, 0.36)	<0.001
*P* for interaction (Age*SES)^b^				0.77	0.80	0.02	0.08	

Footnotes. Significant differences in bold.

a Adjusted for Indigenous and non-English speaking background.

b Interaction test for differential trend in BMI by age compared to most advantaged quintile.

**Figure 1:**
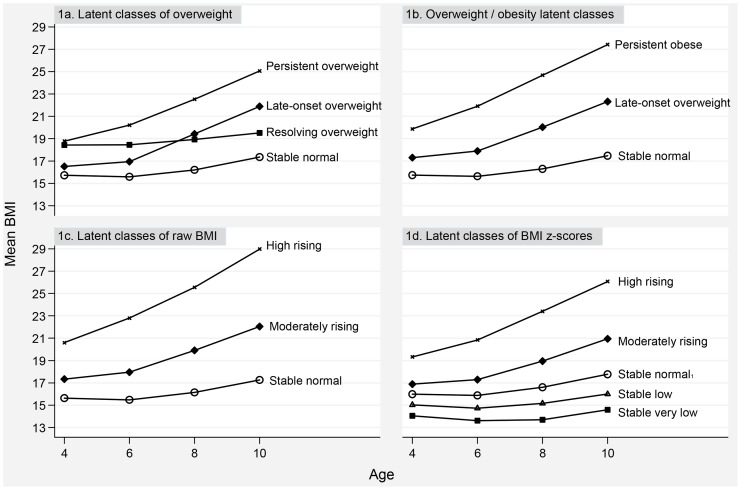
Mean BMI trajectories from age 4–5 to 10–11 years of different latent classes.

Mean BMI trajectories from age 4–5 to 10–11 years are presented for the four separate latent class categorizations in Figures 1a–d. The number of trajectories differed depending on how weight was defined (model fit indices for latent class analyses are summarized in Table S1). All four models identified a ‘stable normal’ BMI category, as well as two distinct trajectories of children with a high BMI: one labelled ‘late-onset overweight’ (or ‘moderately rising’), the other ‘persistent overweight/obese’ (or ‘high rising’). Trajectories based on overweight status (Figure 1a) included a distinct ‘resolving overweight’ category comprising 3% of the population, not identified in the other groupings, characterized by relatively high BMI at age 4 that resolved into normal BMI by late childhood. Finally, trajectories based on BMIz also yielded two trajectories of children with ‘stable low’ or ‘stable very low BMI’ (Figure 1d). Table 2 shows that the prevalence of all high BMI and overweight trajectory classes gradually increased with lowering family and neighbourhood SES quintiles.

**Table 2:** Prevalence of weight trajectory classes by family and neighbourhood socioeconomic status.

	% children in weight trajectory per family SES quintile					% children in weight trajectory per neighbourhood SES quintile				
Latent class	Most advantaged	2^nd^ quintile	3^rd^ quintile	4^th^ quintile	Most disadvantaged	Most advantaged	2^nd^ quintile	3^rd^ quintile	4^th^ quintile	Most disadvantaged
**Trajectories of overweight**										
Persistent overweight	8.9	13.5	14.0	17.1	19.1	11.8	13.9	13.5	15.5	17.3
Late overweight	11.1	11.6	15.0	15.0	16.1	11.8	14.4	12.9	13.8	15.7
Resolving overweight	2.9	2.3	3.0	2.6	2.9	2.8	2.3	2.8	2.9	2.9
*Stable normal weight*	77.0	72.6	68.0	65.2	61.9	73.6	69.5	70.8	67.8	64.2
**Trajectories of overweight and obesity**										
Persistent obese	3.4	7.2	6.6	8.9	11.5	5.3	6.2	6.5	8.1	10.7
Late overweight	15.8	16.6	21.8	22.2	23.1	17.3	20.8	19.1	20.7	21.2
*Stable normal weight*	80.3	76.2	71.6	68.9	65.4	77.4	73.0	74.4	71.2	68.1
**Trajectories of BMI**										
High rising	2.7	4.9	4.2	6.9	8.4	3.8	4.3	4.5	5.5	8.3
Moderately rising	22.2	23.2	28.4	29.8	31.3	24.0	27.0	26.3	28.0	28.9
*Stable normal weight*	75.1	71.9	67.3	63.3	69.3	72.2	68.7	69.2	66.5	62.8
**Trajectories of BMI z-scores**										
High rising	6.5	10.8	10.9	13.9	16.2	8.5	10.6	10.6	12.6	15.0
Moderately rising	26.9	25.0	29.5	30.7	30.7	27.3	28.9	29.2	27.9	29.3
*Stable normal weight*	40.9	36.5	35.6	33.2	31.0	39.0	35.1	37.0	35.3	31.5
Stable low	20.6	21.7	19.2	18.7	17.4	20.4	20.1	19.3	19.5	18.8
Stable very low	5.1	6.1	4.8	3.5	4.8	4.9	5.3	3.9	4.7	5.4

For our primary classification (Figure 1a), Table 3 shows that higher levels of family disadvantage were associated with progressively higher risks of persistent and late-onset overweight (model 1: *P* for trend <0.001 for both trajectories; e.g. OR_persistent_ = 2.51, 95% CI: 1.83, 3.43). Additional adjustment for birth weight and parental BMI as potential mediators of the SES-BMI association (Model 2) and for neighbourhood disadvantage (Model 3) attenuated but did not eliminate the effect estimates. Attenuation from model 1 to 2 was mostly due to parental BMI, whereas birth weight explained little of the SES-BMI relationship. ORs for neighbourhood disadvantage were similar in pattern to family SES but smaller (Model 1), attenuated to non-significance with parent BMI and birth weight (Model 2), and virtually disappeared with the addition of family SES (Model 3). SES did not predict resolving overweight (*P* for trend = 0.38 for family SES, and 0.40 for neighbourhood SES).

**Table 3:** Association between socioeconomic status and longitudinal overweight trajectories from age 4–5 to 10–11 years.

SES quintiles	OR for overweight trajectory per family SES quintile^a^			OR for overweight trajectory per neighbourhood SES quintile^a^			
	Persistent (n≈723)	Late-onset (n≈683)	Resolving (n≈134)	Persistent (n≈723)	Late-onset (n≈683)	Resolving (n≈134)	
	OR (95% CI)	OR (95% CI)	OR (95% CI)	OR (95% CI)	OR (95% CI)	OR (95% CI)	
**Model 1: adjusted for confounders (Indigenous status and non-English speaking background)**							
2^nd^ quintile	**1.61** (1.19, 2.17)	1.11 (0.80, 1.55)	0.86 (0.45, 1.65)	1.29 (0.89, 1.87)	1.32 (0.96, 1.83)	0.87 (0.44, 1.71)	
3^rd^ quintile	**1.78** (1.30, 2.44)	**1.54** (1.13, 2.09)	1.18 (0.67, 2.11)	1.23 (0.87, 1.74)	1.16 (0.81, 1.64)	1.06 (0.56, 2.01)	
4^th^ quintile	**2.28 (**1.69, 3.09)	**1.60** (1.15, 2.23)	1.08 (0.58, 2.00)	**1.50** (1.06, 2.12)	1.31 (0.96, 1.80)	1.15 (0.59, 2.23)	
Most disadvantaged	**2.51** (1.83, 3.43)	**1.78** (1.28, 2.49)	1.24 (0.65, 2.36)	**1.66** (1.18, 2.33)	**1.55** (1.12, 2.13)	1.19 (0.65, 2.19)	
*P* for trend	<0.001	<0.001	0.38	0.002	0.02	0.40	
**Model 2: model 1 additionally adjusted for birth weight and parental BMI**							
2^nd^ quintile	**1.42** (1.04, 1.93)	1.02 (0.73, 1.43)	0.79 (0.41, 1.52)	1.22 (0.84, 1.78)	1.27 (0.92, 1.76)	0.85 (0.43, 1.69)	
3^rd^ quintile	**1.44** (1.05, 1.98)	1.34 (0.97, 1.84)	1.04 (0.58, 1.87)	1.09 (0.77, 1.54)	1.07 (0.75, 1.53)	1.01 (0.52, 1.90)	
4^th^ quintile	**1.75** (1.28, 2.41)	1.35 (0.95, 1.91)	0.93 (0.50, 1.75)	1.20 (0.85, 1.70)	1.13 (0.83, 1.56)	1.02 (0.52, 1.98)	
Most disadvantaged	**1.90** (1.36, 2.67)	**1.48** (1.02, 2.14)	1.07 (0.55, 2.09)	1.33 (0.95, 1.86)	1.34 (0.97, 1.85)	1.05 (0.57, 1.94)	
*P* for trend	<0.001	0.02	0.71	0.12	0.19	0.73	
**Model 3: model 2 with mutual adjustment for family and neighbourhood SES**							
2^nd^ quintile	**1.41** (1.02, 1.93)	1.01 (0.73, 1.44)	0.78 (0.40, 1.53)	1.12 (0.76, 1.65)	1.20 (0.87, 1.66)	0.85 (0.43, 1.70)	
3^rd^ quintile	**1.42** (1.02, 1.98)	1.31 (0.96, 1.84)	1.04 (0.57, 1.89)	0.96 (0.66, 1.38)	0.98 (0.68, 1.42)	1.00 (0.51, 1.94)	
4^th^ quintile	**1.73** (1.23, 2.44)	1.33 (0.92, 1.91)	0.92 (0.48, 1.74)	1.03 (0.71, 1.49)	1.02 (0.73, 1.43)	1.01 (0.50, 2.01)	
Most disadvantaged	**1.88** (1.30, 2.72)	**1.47** (1.01, 2.18)	1.05 (0.51, 2.13)	1.09 (0.76, 1.58)	1.18 (0.84, 1.65)	1.03 (0.54, 1.97)	
*P* for trend	<0.001	0.006	0.45	0.31	0.35	0.62	

Footnotes. Significant ORs in bold.

a Reference categories: “Stable normal weight” (n≈3409) for trajectories; “Most advantaged quintile” for SES.

**Figure 2:**
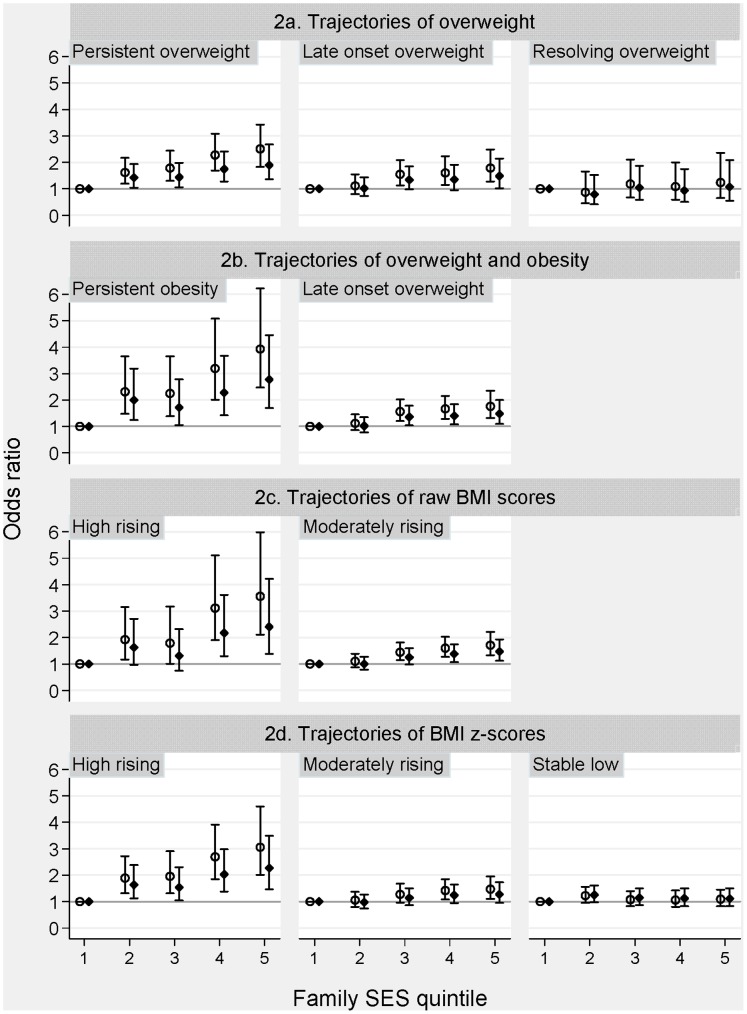
Association between family socioeconomic status and weight trajectories from age 4–5 to age 10–11 years based on different BMI classifications. Footnotes Figure 2. Hollow-Circles (○) represent ORs adjusted for confounders (Indigenous status and non-English speaking background) and diamond symbols (♦) represent ORs additionally adjusted for birth weight and parental BMI. Bars represent 95% CI.

Figure 2 graphically displays the associations between SES and the identified trajectories for all four latent class categorizations; full analyses for trichotomized BMI (Figure 2b), BMI raw scores (Figure 2c) and BMIz (Figure 2d) are detailed in tables S2, S3 and S4. All showed similar patterns of large socioeconomic inequalities in rising BMI across childhood. Most strikingly, Figure 2b shows that the most disadvantaged children were nearly four times more likely to be in the ‘persistent obesity’ trajectory than the most advantaged (OR = 3.92, 95% CI: 2.47, 6.22); recall that this trajectory was characterized by an average BMI of around 20 at age 4–5 increasing to 27 at age 10–11 years. Noteworthy is also the lack of a socioeconomic gradient in childhood trajectories of stable (very) low BMI.

## Discussion

In this contemporary population-based cohort of Australian children, we showed that socioeconomic differences in high BMI already present at age 4–5 years not only persisted but had more than doubled by age 10–11 years, confirming and extending findings from other countries [14], [15]. These results were robust across different longitudinal BMI classifications and, collectively, suggest that childhood has now become the critical period during which the SES-BMI association is established. The most striking socioeconomic gradients were seen for children whose high BMI had already developed by the preschool years then rapidly increased; however, disadvantaged children were also more likely to follow a trajectory of overweight that first developed in middle childhood.

Intriguingly, this striking pattern of entrenching socioeconomic inequalities across middle childhood appeared to reflect two complementary longitudinal influences: decreasing mean BMI in the more advantaged, coupled with simultaneously worsening rates of overweight in the more disadvantaged. Thus, the high mean BMI of 0.47 evident at age 4–5 years in the most advantaged quintile lessened steadily with age to reach 0.2 by 10–11 years – approaching mean levels not seen since the normative populations of the 1960–90s [33]. Simultaneously, at the extreme of the BMI distribution, risks of persistent and developing overweight and obesity in the most disadvantaged children were significantly higher as opposed to socially advantaged children. We suggest that examining factors underlying the decreases in mean BMI among children of the most advantaged families may prove as informative for future prevention and intervention strategies as focusing on risk factors for overweight among socially-disadvantaged children.

A further novel finding [17]–[20] was that, while both family and neighbourhood social gradients were apparent in these trajectories, the neighbourhood SES associations were smaller, had plateaued by age 6–7 years, and attenuated fully once family SES was accounted for. This contrasts with our earlier cross-sectional findings for these same children at age 4 years [32], suggesting that neighbourhood characteristics such as safety and availability of playgrounds and parks – known to be associated with overweight in children [23] – may become relatively less important once children commence school. School and neighbourhood attributes relating to obesity are not well captured in the LSAC dataset, but we could speculate that attributes such as commuting [43], physical activity at schools, and the school food environment (e.g. fat percentage and calories per meal, availability of vending machines) [44] might be less socially patterned than the neighbourhood characteristics contributory to early childhood BMI [45]. Conversely, although availability of fast food and convenience stores is socially patterned and known to predict overweight development [46], this might not play a substantial role until children become slightly older.

This paper examined inequalities in the typical BMI trajectories Australian children follow from age 4–5 years. Nonetheless, we did adjust our models for two factors – child birth weight and parental BMI – for which there was strong pre-existing evidence of a link with BMI. For example, parental anthropometrics could influence childhood overweight via foetal over-nutrition [24], [26] as well as shared postnatal obesogenic environmental and lifestyle factors, such as diet quality, physical activity and sedentary behaviours [21], [22]. However, the fact that family SES continued to strongly predict the most concerning BMI trajectories even *after* adjusting for birth weight (which contributed very little to the models) and parental BMI illustrates the importance of prioritizing research into the causal, and potentially modifiable, mechanisms underlying these associations.

Contrary to our initial hypothesis and despite the mean BMI improvements in advantaged children, we found no evidence for a socioeconomic gradient in resolving overweight. Overweight in young children may resolve due to changes in lifestyle or eating habits. However, the lack of a social gradient in this trajectory suggests that resolution may be more genetically than environmentally or behaviourally driven. ‘Resolving overweight’ may also partly reflect a natural phenomenon of ‘regression to the mean’ where a period of rapid growth tends to be followed by a period of slower growth and normalisation in relation to peers [29]. Although representing only 3% of the LSAC cohort, these children comprise a sizable proportion (16%) of those who were initially overweight or obese. Further research into this potentially-important group could therefore offer valuable insights to inform prevention and treatment approaches. Finally, neither family nor neighbourhood SES was associated with childhood trajectories of low BMI in our study, suggesting that the socioeconomic inequalities in underweight – still strong in developing countries [47] – may be weakening in primary school-aged children in wealthy countries.

Strengths of the present study were its large population-based sample, repeatedly measured BMI, the availability of validated measures of both family and neighbourhood SES, and the robustness of findings to a variety of BMI classifications used to model trajectories. The different BMI classifications yielded a large degree of congruence with only some points of difference, which collectively supported our conclusions about social inequalities across a range of different patterns of BMI and overweight. While some BMI trajectories mirrored those previously reported [17]–[20], the resolving overweight trajectory was novel. This suggests that future research might benefit from similar empirical approaches to capture the changing shape of the obesity epidemic as it continues to rapidly evolve.

Limitations included the use of BMI as a proxy measure of adiposity because it may misclassify certain children, particularly those with less severe overweight [48]. Nevertheless, even these children appear to have a greater risk of adverse health outcomes than those with lower BMI [48]. Families from disadvantaged areas are relatively underrepresented in LSAC but, by using sampling weights, we accounted at least partially for this imbalance. Further, despite the very high retention rates at every wave, data on child BMI were more often missing in these families. This may have lowered the prevalence estimates for the detrimental overweight trajectories because the prevalence is generally higher in the more disadvantaged group. However, the spread of SES was sufficiently wide to observe substantial socioeconomic inequalities.

In summary, we suggest that childhood has now become the critical period during which the SES-BMI association is established. Clearly, targeting children with early overweight *and* low socioeconomic background – particularly those from socially disadvantaged families – must be a top intervention priority. Nonetheless, as vulnerability to unhealthy weight gain increases incrementally with each quintile further from the most advantaged, only whole-society approaches will eliminate overweight-associated morbidity. This supports an approach of universal health actions implemented with a scale and an intensity proportionate to the level of disadvantage [13].

## Supporting Information

Table S1 (DOC)

Table S2 (DOC)

Table S3 (DOC)

Table S4 (DOC)
