# Correction: Family and Neighbourhood Socioeconomic Inequalities in Childhood Trajectories of BMI and Overweight: Longitudinal Study of Australian Children

**DOI:** 10.1371/annotation/f7e5e1f3-77f6-4c56-b0ba-53b54a86df14

**Published:** 2013-07-31

**Authors:** Pauline W. Jansen, Fiona K. Mensah, Jan M. Nicholson, Melissa Wake

Institutional affiliation number 1 for all authors is incorrect. The correct affiliation is: 1, Murdoch Children’s Research Institute, Melbourne, Australia.

