# Supplementary material for: Family and Neighbourhood Socioeconomic Inequalities in Childhood Trajectories of BMI and Overweight: Longitudinal Study of Australian Children
Source: PLoS One. 2013 Jul 23;8(7):e69676. doi: 10.1371/journal.pone.0069676 (PMC3720589; doi:10.1371/journal.pone.0069676)
Supplement: Table S1 — (DOC) [file pone.0069676.s001.doc]

Table S1.

|  | Models based on BMI categories^a^ | | | | |  | Models based on BMI scores^a^ | | | | |
| --- | --- | --- | --- | --- | --- | --- | --- | --- | --- | --- | --- |
| No. of classes | Dichotomized BMI | |  | Trichotomized BMI | |  | BMI raw scores | |  | BMI z-scores | |
|  | BIC | *P* |  | BIC | *P* |  | BIC | *P* |  | BIC | *P* |
| 1 | 18729 | -- |  | 23424 | -- |  | 82065 | -- |  | 50466 | -- |
| 2 | 13735 | <0.0001 |  | 18156 | <0.0001 |  | 73929 | <0.0001 |  | 43938 | <0.0001 |
| 3 | 13526 | <0.0001 |  | **17139** | **0.0005** |  | **70301** | **0.04** |  | 40542 | <0.0001 |
| 4 | **13451** | **<0.0001** |  | 17037 | 0.67 |  | 68247 | 0.12 |  | 38833 | 0.008 |
| 5 | 13493 | 0.50 |  |  |  |  |  |  |  | **37933** | **0.0001** |
| 6 |  |  |  |  |  |  |  |  |  | 37563 | 0.09 |
