# Supplementary material for: Family and Neighbourhood Socioeconomic Inequalities in Childhood Trajectories of BMI and Overweight: Longitudinal Study of Australian Children
Source: PLoS One. 2013 Jul 23;8(7):e69676. doi: 10.1371/journal.pone.0069676 (PMC3720589; doi:10.1371/journal.pone.0069676)
Supplement: Table S2 — (DOC) [file pone.0069676.s002.doc]

Table S2.

|  | OR for overweight/obesity trajectory per **family SES** quintile^a^ | |  | OR for overweight/obesity trajectory per **neighbourhood SES** quintile^a^ | |
| --- | --- | --- | --- | --- | --- |
|  | Persistent obese (n≈371) | Late-onset overweight (n≈985) |  | Persistent obese (n≈371) | Late-onset overweight (n≈985) |
| **SES quintiles** | OR (95% CI) | OR (95% CI) |  | OR (95% CI) | OR (95% CI) |
| **Model 1: adjusted for confounders (Indigenous status and non-English speaking background)** | | | | | |
| 2^nd^ quintile | **2.31** (1.47, 3.65 | 1.11 (0.86, 1.45 |  | 1.33 (0.74, 2.39 | 1.30 (0.97, 1.73 |
| 3^rd^ quintile | **2.25** (1.38, 3.65 | **1.56** (1.20, 2.02 |  | 1.36 (0.78, 2.35 | 1.17 (0.89, 1.54 |
| 4^th^ quintile | **3.19** (2.01, 5.07 | **1.66** (1.28, 2.15 |  | **1.82** (1.06, 3.11 | **1.33** (1.01, 1.76 |
| Most disadvantaged | **3.92** (2.47, 6.22 | **1.76** (1.32, 2.35 |  | **2.30** (1.36, 3.88 | **1.39** (1.05, 1.83 |
| *P* for trend | <0.001 | <0.001 |  | <0.001 | 0.03 |
| **Model 2: model 1 additionally adjusted for birth weight and parental BMI** | | | | |  |
| 2^nd^ quintile | **1.99** (1.24, 3.19) | 1.02 (0.78, 1.34) |  | 1.24 (0.68, 2.25) | 1.25 (0.94, 1.67) |
| 3^rd^ quintile | **1.71** (1.05, 2.78) | **1.36** (1.04, 1.78) |  | 1.18 (0.68, 2.04) | 1.08 (0.82, 1.43) |
| 4^th^ quintile | **2.28** (1.42, 3.67) | **1.40** (1.07, 1.83) |  | 1.39 (0.82, 2.38) | 1.15 (0.87, 1.52) |
| Most disadvantaged | **2.77** (1.69, 4.54) | **1.48** (1.09, 2.00) |  | **1.76** (1.05, 2.96) | 1.20 (0.91, 1.59) |
| *P* for trend | <0.001 | 0.001 |  | 0.02 | 0.39 |
| **Model 3: model 2 with mutual adjustment for family and neighbourhood SES** | | | | |  |
| 2^nd^ quintile | **1.93** (1.19, 3.13) | 1.02 (0.77, 1.35) |  | 1.14 (0.62, 2.09) | 1.17 (0.88, 1.55) |
| 3^rd^ quintile | 1.61 (0.96, 2.70) | 1.35 (1.03, 1.78) |  | 1.00 (0.56, 1.79) | 0.98 (0.73, 1.31) |
| 4^th^ quintile | **2.12** (1.25, 3.58) | 1.40 (1.06, 1.85) |  | 1.16 (0.65, 2.06) | 1.02 (0.76, 1.36) |
| Most disadvantaged | **2.54** (1.46, 4.41) | **1.48** (1.08, 2.04) |  | 1.39 (0.78, 2.45) | 1.04 (0.78, 1.39) |
| *P* for trend | <0.001 | <0.001 |  | 0.07 | 0.71 |
