# Supplementary material for: Family and Neighbourhood Socioeconomic Inequalities in Childhood Trajectories of BMI and Overweight: Longitudinal Study of Australian Children
Source: PLoS One. 2013 Jul 23;8(7):e69676. doi: 10.1371/journal.pone.0069676 (PMC3720589; doi:10.1371/journal.pone.0069676)
Supplement: Table S3 — (DOC) [file pone.0069676.s003.doc]

Table S3.

|  | OR for raw BMI trajectory per **family SES** quintile^a^ | |  | OR for raw BMI trajectory per **neighbourhood SES** quintile^a^ | |
| --- | --- | --- | --- | --- | --- |
|  | High rising (n≈267) | Moderately rising (n≈1336) |  | High rising (n≈267) | Moderately rising (n≈1336) |
| **SES quintiles** | OR (95% CI) | OR (95% CI) |  | OR (95% CI) | OR (95% CI) |
| **Model 1: adjusted for confounders (Indigenous status and non-English speaking background)** | | | | | |
| 2^nd^ quintile | **1.92** (1.17, 3.16) | 1.10 (0.87, 1.39) |  | 1.28 (0.63, 2.59) | 1.21 (0.93, 1.57) |
| 3^rd^ quintile | **1.79** (1.01, 3.17) | **1.44** (1.14, 1.81) |  | 1.32 (0.68, 2.54) | 1.16 (0.89, 1.52) |
| 4^th^ quintile | **3.12** (1.90, 5.11) | **1.60** (1.27, 2.03) |  | 1.73 (0.91, 3.30) | **1.30** (1.02, 1.66) |
| Most disadvantaged | **3.56** (2.11, 5.98) | **1.71** (1.33, 2.21) |  | **2.53** (1.36, 4.70) | **1.39** (1.08, 1.78) |
| *P* for trend | <0.001 | <0.001 |  | 0.001 | 0.01 |
| **Model 2: model 1 additionally adjusted for birth weight and parental BMI** | | | | |  |
| 2^nd^ quintile | 1.62 (0.97, 2.71) | 1.00 (0.79, 1.27) |  | 1.19 (0.58, 2.42) | 1.16 (0.89, 1.51) |
| 3^rd^ quintile | 1.31 (0.74, 2.32) | 1.25 (0.98, 1.59) |  | 1.13 (0.59, 2.16) | 1.07 (0.82, 1.40) |
| 4^th^ quintile | **2.17** (1.30, 3.61) | **1.38** (1.07, 1.74) |  | 1.28 (0.68, 2.42) | 1.12 (0.87, 1.43) |
| Most disadvantaged | **2.41** (1.38, 4.22) | **1.47** (1.12, 1.93) |  | **1.89** (1.03, 3.49) | 1.20 (0.93, 1.55) |
| *P* for trend | 0.002 | 0.001 |  | 0.02 | 0.24 |
| **Model 3: model 2 with mutual adjustment for family and neighbourhood SES** | | | | |  |
| 2^nd^ quintile | 1.57 (0.93, 2.64) | 0.99 (0.78, 1.28) |  | 1.12 (0.54, 2.32) | 1.10 (0.84, 1.43) |
| 3^rd^ quintile | 1.24 (0.68, 2.24) | 1.24 (0.96, 1.60) |  | 0.97 (0.48, 1.96) | 0.98 (0.74, 1.31) |
| 4^th^ quintile | **1.98** (1.12, 3.50) | **1.36** (1.05, 1.77) |  | 1.10 (0.55, 2.20) | 1.01 (0.77, 1.31) |
| Most disadvantaged | **2.17** (1.15, 4.09) | **1.47** (1.10, 1.96) |  | 1.51 (0.76, 2.99) | 1.06 (0.81, 1.38) |
| *P* for trend | 0.004 | <0.001 |  | 0.07 | 0.50 |
