# Supplementary material for: Family and Neighbourhood Socioeconomic Inequalities in Childhood Trajectories of BMI and Overweight: Longitudinal Study of Australian Children
Source: PLoS One. 2013 Jul 23;8(7):e69676. doi: 10.1371/journal.pone.0069676 (PMC3720589; doi:10.1371/journal.pone.0069676)
Supplement: Table S4 — (DOC) [file pone.0069676.s004.doc]

Table S4.

|  | OR for BMIz trajectory per **family SES** quintile^a^ | | |  | OR for BMIz trajectory per **neighbourhood SES** quintile^a^ | | |
| --- | --- | --- | --- | --- | --- | --- | --- |
|  | High rising (n≈574) | Moderately rising (n≈1415) | Stable low^b^ (n≈1208) |  | High rising (n≈574) | Moderately rising (n≈1415) | Stable low^b^ (n≈1208) |
| **SES quintiles** | OR (95% CI) | OR (95% CI) | OR (95% CI) |  | OR (95% CI) | OR (95% CI) | OR (95% CI) |
| **Model 1: adjusted for confounders (Indigenous status and non-English speaking background)** | | | | | | | |
| 2^nd^ quintile | **1.89** (1.31, 2.72) | 1.05 (0.80, 1.37) | 1.22 (0.95, 1.56) |  | 1.48 (0.90, 2.42) | 1.20 (0.90, 1.59) | 1.14 (0.88, 1.49) |
| 3^rd^ quintile | **1.95** (1.31, 2.90) | 1.27 (0.96, 1.67) | 1.07 (0.82, 1.40) |  | 1.39 (0.88, 2.18) | 1.15 (0.87, 1.52) | 0.99 (0.77, 1.28) |
| 4^th^ quintile | **2.69** (1.85, 3.91) | **1.42** (1.08, 1.85) | 1.06 (0.80, 1.42) |  | **1.78** (1.14, 2.78) | 1.15 (0.88, 1.51) | 1.10 (0.84, 1.43) |
| Most disadvantaged | **3.05** (2.01, 4.60) | **1.47** (1.10, 1.95) | 1.09 (0.82, 1.45) |  | **2.19** (1.41, 3.40) | **1.33** (1.02, 1.74) | 1.19 (0.92, 1.53) |
| *P* for trend | <0.001 | 0.001 | 0.87 |  | <0.001 | 0.08 | 0.31 |
| **Model 2: model 1 additionally adjusted for birth weight and parental BMI** | | | |  |  |  |  |
| 2^nd^ quintile | **1.63** (1.12, 2.38) | 0.97 (0.74, 1.27) | 1.25 (0.98, 1.61) |  | 1.39 (0.84, 2.29) | 1.16 (0.87, 1.55) | 1.15 (0.88, 1.51) |
| 3^rd^ quintile | **1.54** (1.04, 2.30) | 1.14 (0.86, 1.50) | 1.14 (0.86, 1.50) |  | 1.23 (0.78, 1.94) | 1.08 (0.82, 1.43) | 1.02 (0.78, 1.32) |
| 4^th^ quintile | **2.03** (1.38, 2.98) | 1.24 (0.94, 1.64) | 1.12 (0.83, 1.50) |  | 1.40 (0.89, 2.19) | 1.03 (0.78, 1.35) | 1.16 (0.89, 1.52) |
| Most disadvantaged | **2.27** (1.47, 3.49) | 1.28 (0.95, 1.73) | 1.11 (0.82, 1.51) |  | **1.73** (1.12, 2.67) | 1.18 (0.90, 1.55) | 1.23 (0.95, 1.59) |
| *P* for trend | <0.001 | 0.03 | 0.73 |  | 0.02 | 0.48 | 0.16 |
| **Model 3: model 2 with mutual adjustment for family and neighbourhood SES** | | | |  |  |  |  |
| 2^nd^ quintile | **1.58** (1.07, 2.33) | 0.97 (0.73, 1.27) | 1.23 (0.95, 1.58) |  | 1.27 (0.76, 2.12) | 1.11 (0.84, 1.48) | 1.12 (0.85, 1.48) |
| 3^rd^ quintile | 1.46 (0.96, 2.21) | 1.13 (0.85, 1.51) | 1.10 (0.82, 1.46) |  | 1.06 (0.65, 1.71) | 1.02 (0.76, 1.36) | 0.98 (0.75, 1.28) |
| 4^th^ quintile | **1.90** (1.25, 2.89) | 1.24 (0.93, 1.64) | 1.07 (0.78, 1.46) |  | 1.18 (0.73, 1.91) | 0.95 (0.71, 1.27) | 1.13 (0.85, 1.49) |
| Most disadvantaged | **2.10** (1.31, 3.37) | 1.28 (0.91, 1.75) | 1.05 (0.77, 1.45) |  | 1.38 (0.86, 2.23) | 1.08 (0.81, 1.43) | 1.21 (0.92, 1.58) |
| *P* for trend | <0.001 | 0.006 | 0.82 |  | 0.07 | 0.64 | 0.21 |
